# Zirconium Phosphate-Pillared Zeolite MCM-36 for Green Production of γ-Valerolactone from Levulinic Acid via Catalytic Transfer Hydrogenation

**DOI:** 10.3390/molecules29163779

**Published:** 2024-08-09

**Authors:** Pan Hou, Haopeng Su, Keyan Jin, Qiang Li, Wenfu Yan

**Affiliations:** State Key Laboratory of Inorganic Synthesis and Preparative Chemistry, College of Chemistry, Jilin University, Changchun 130012, China; houpan21@mails.jlu.edu.cn (P.H.); hf_suhaopeng@outlook.com (H.S.); jinky23@mails.jlu.edu.cn (K.J.); liqiang23@mails.jlu.edu.cn (Q.L.)

**Keywords:** biomass, levulinic acid, zeolites, catalytic transfer hydrogenation, MCM-22

## Abstract

γ-valerolactone (GVL), derived from biomass, is a crucial platform compound for biofuel synthesis and various industrial applications. Current methods for synthesizing GVL involve expensive catalysts and high-pressure hydrogen, prompting the search for greener alternatives. This study focuses on a novel zirconium phosphate (ZrP)-pillared zeolite MCM-36 derivative catalyst for converting levulinic acid (LA) to GVL using alcohol as a hydrogen source. The incorporation of ZrP significantly contributes to mesoporosity and greatly enhances the acidity of the catalysts. Additionally, we employed ^31^P MAS NMR to comprehensively investigate the influence of phosphorus species on both the acidity and the catalytic conversion of LA to GVL. By adjusting the Zr-to-P ratios, we synthesized catalysts with enhanced acidity, achieving high conversion of LA and selectivity for GVL. The catalyst exhibited high recyclability, showing only minor deactivation over the course of five cycles. Furthermore, the catalyst was successfully applied to the one-pot conversion of furfural to GVL, showcasing its versatility in biomass conversion. This study highlights the potential of the MCM-ZrP1 catalyst for sustainable biomass conversion and offers insights for future research in renewable energy technologies.

## 1. Introduction

In recent decades, unprecedented environmental problems, including emissions of greenhouse gases and pollutants such as SO_x_ and NO_x_, have resulted from the rapid global consumption of fossil fuels [1,2]. A major challenge for sustainable development in the 21st century is transitioning from fossil fuel-based industries to renewable energy-based industries [3,4]. Upgrading biomass, such as lignocellulosic materials, into high value-added products has garnered significant attention [5]. γ-valerolactone (GVL), a biomass-derived platform compound, is considered an important intermediate in biofuel synthesis and has received much attention [6]. Due to its excellent physicochemical properties, GVL is also widely used as a polymer monomer and as a green solvent [7,8].

Currently, GVL is primarily prepared by the direct hydrogenation of LA and its esters using hydrogen gas [9,10]. Numerous precious [11,12,13] and non-precious [14,15,16] metal catalysts are employed in this reaction. However, the main drawbacks of these catalytic hydrogenation systems are the high cost of catalysts and the safety concerns associated with high-pressure hydrogen (e.g., purchase, transport, safety hazards, and expensive infrastructure) [17]. As the principles of green chemistry and sustainability gain importance, novel catalysts and technologies need to be developed to simplify the preparation process [18,19].

Compared to traditional hydrogenation with molecular hydrogen, the catalytic transfer hydrogenation (CTH) strategy offers a more compelling alternative. In CTH reactions, using an indirect hydrogen source, such as formic acid (FA) or alcohols, mitigates many problems associated with high-pressure molecular hydrogen, thereby enhancing the sustainability of industrial processes.

Guo et al. [20] first reported a novel route to convert various biomass-derived oxygenates into GVL using FA as a hydrogen source. FA can in situ generate molecular hydrogen for the hydrogenation of LA by decomposing over metal catalysts [21]. While formic acid remains a viable option for the CTH reaction, its irritating and corrosive properties pose safety concerns. In this context, the recently developed CTH reaction system using alcohols as a hydrogen source presents a more favorable alternative for GVL production [22]. In this system, alcohols act as both solvents and hydrogen donors. The current research explores the potential of various types of heterogeneous solid acids containing Lewis and Brønsted acids for the conversion of biomass into high-value products.

In pioneering work, Chia and Dumesic reported the first catalytic transfer hydrogenation process using zirconium oxide (ZrO_2_) as a catalyst for converting LA and its esters to GVL [23]. Furthermore, Sun et al. significantly advanced the CTH reaction system by introducing zirconium hydroxide as an efficient catalyst [24]. Inspired by these works, more materials have been developed, such as metal–organic hybrid materials [25], metal–organic frameworks [26], and zeolites [18,27,28]. Despite the promising advancements achieved with the CTH reaction system, developing even more efficient and recyclable catalyst systems remains an urgent research priority. Ideally, these catalysts should function under mild reaction conditions for optimal sustainability.

Zeolites are a class of solid acid catalysts that hold immense promise for biomass conversion due to their unique properties. Notably, zeolites possess a remarkably high specific surface area, providing ample space for reactant molecules to interact with the catalyst [29]. They also exhibit tunable acidity, allowing for precise tailoring of the catalyst to specific reactions and optimizing its performance [30]. Additionally, zeolites demonstrate excellent surface diffusion capacity, facilitating the movement of reactants and products within the catalyst pores.

The zeolite frameworks are generally very stable at high temperatures, a crucial requirement for many catalytic reactions. This thermal stability ensures the catalyst’s effectiveness under demanding reaction conditions. Zeolite catalysts can often be easily separated from the reaction mixture and regenerated for further use, reducing operating costs and minimizing waste generation. These combined properties contribute to their high catalytic activity and efficiency [31], making them highly promising for biomass catalysis. Zeolites doped with heteroatoms that exhibit both Lewis and Brønsted acidity, particularly Zr(IV), Hf(IV), and Sn(IV), have garnered significant attention due to their exceptional catalytic activity in converting LA to GVL [32,33]. This research area has been actively explored using various zeolites, including Zr-Beta, Al-MFI (mixed zeolite) [34], Hf-Al-USY [35], Zr-Al-Beta [27], and heteropolyacid-supported Zr-Beta [18].

Zeolite MCM-22, first reported by Mobil in 1990, is the reference material for the **MWW** zeotype, a unique zeolitic framework code granted by the International Zeolite Association (IZA). In addition to MCM-22, the **MWW** family includes several other zeolites, such as SSZ-25 [36], ERB-1 [37], and ITQ-1 [38], each contributing to the diverse applications and characteristics of this topology. Hydrothermal synthesis first produces a lamellar precursor, MCM-22(P). Calcination then condenses the silica hydroxyl groups between layers, transforming it into the final three-dimensional MCM-22 structure [39]. This structure features two independent, non-intersecting channel systems. One system consists of two-dimensional (2D), ten-membered ring (10-MR) sinusoidal channels within the layers (intralamellar). The other system comprises interconnected channels between the layers (interlamellar) that include larger 12-MR supercages [40].

Before calcination, the MCM-22 layers can be separated by surfactant swelling, and the interlayer spaces can be partially pillared with thermally stable inorganic columns [41]. In this scenario, the 10-MR sinusoidal channels of the **MWW** zeolite layer remain unaltered, while the 12-MR supercages that form after the linkage of the **MWW** layer are not formed after the pillaring process. Instead, this process produces a novel mesoporous cavity with pore sizes between 2.5 nm and 3.0 nm due to the effective intercalation of the silica columns. Pillars formed from oxides other than silica can provide additional active sites, such as BaO-Al_2_O_3_, Al_2_O_3_-SiO_2_, MgO-Al_2_O_3_-SiO_2_, and BaO-Al_2_O_3_-SiO_2_. These **MWW** columnar materials are acid–base bifunctional materials containing enhanced Lewis and Brønsted acidity located in the interlayer space between the zeolite layers [42].

In this study, we developed a zirconium phosphate (ZrP)-pillared zeolite MCM-36 derivative, MCM-ZrPx (where x represents the feeding ratio of Zr to P). We investigated its structural and acidic properties and applied this catalyst to the catalytic conversion of LA to GVL, using environmentally friendly alcohols as the hydrogen source. By adjusting different ratios of Zr to P, we optimized the structures and achieved high catalytic conversion and GVL selectivity. Additionally, we explored different materials in depth to study the composition of their active components and determine the active substance composition.

## 2. Results and Discussion

### 2.1. Characterization of Catalysts

**Scheme 1:**
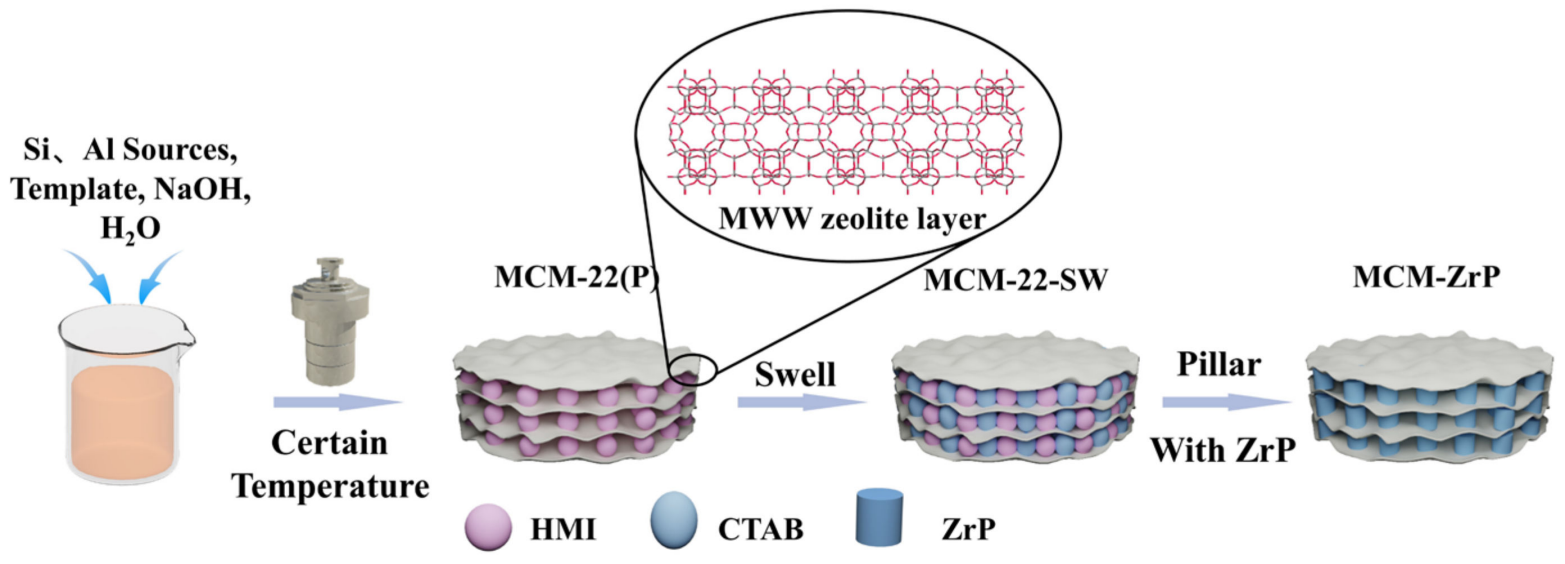
Synthetic procedure of MCM-ZrPx and schematic diagram of the MWW zeolite layer.

The catalyst was synthesized via a two-step process, as shown in Scheme 1. First, MCM-22 was prepared through hydrothermal synthesis. The obtained material was then swelled in the presence of a surfactant, followed by pillaring with zirconium phosphate (ZrP). Before catalytic evaluation, all catalysts were activated by ammonium (NH₄^+^) ion exchange to achieve an H-type zeolite form and then underwent calcination. The schematic diagram of the MWW zeolite is also provided in Scheme 1.

**Figure 1:**
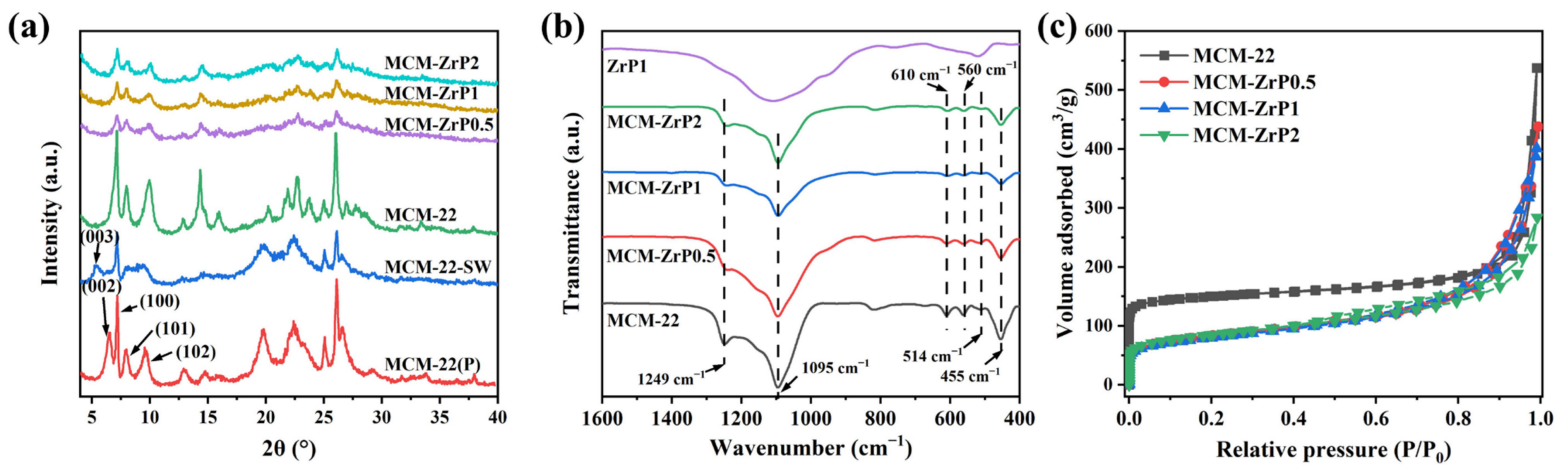
(**a**) XRD patterns of MCM-22(P), MCM-22-SW, MCM-22, and MCM-ZrP catalysts; (**b**) FT-IR spectra; and (**c**) N_2_ adsorption−desorption isotherms of MCM-22 and different MCM-ZrPx catalysts, where x represents the feeding molar ratio of Zr to P.

The X-ray diffraction (XRD) patterns (Figure 1) of MCM-22 (P), MCM-22 after swelling, and MCM-22 after ZrP pillaring show excellent agreement with previously reported data in the literature [43]. The diffraction peak of MCM-22 (P) appearing at about 6.6° before calcination corresponds to the interlayer reflection (002), attributed to alignment perpendicular to the *c*-direction [44]. Additionally, the sample shows two strong diffractions due to the (101) and (102) reflections, which match well with the layered precursor of the **MWW** topology. When the precursor was calcined at 540 °C for 6 h to remove the organic structure-directing agent, interlayer silanol dehydration and condensation occurred, causing the (002) (100) reflections to disappear or shift to higher angles.

As shown in Figure 1, the expansion of all MCM-ZrP materials along the crystallographic *c*-axis induces a slight displacement of the *hk1* reflection, while the *hk0* reflection remains unchanged. For example, the positions of the prominent narrow peaks at 2θ = 7.2°, 25.1°, and 26.1° are not affected, demonstrating that post-treatment did not alter the internal structure of individual zeolite layers [45]. In contrast to MCM-22, MCM-ZrP exhibits a diminished layer arrangement in the third dimension along the *c*-axis direction. This is confirmed by the presence of broad bands in the 2θ range at 8–10°. As a result, the material exhibits a 2D **MWW** topology and a partially delaminated structure.

We then used Fourier transform infrared (FT-IR) spectroscopy (Figure 1b) to investigate the characteristic functional groups in the material. Peaks at 560 and 610 cm^−1^, corresponding to the framework vibrations of the double six-membered ring (D6R) units, are characteristic of the **MWW** topology [46]. Other peaks at 454, 800, and 1095 cm^−1^, characteristic of tetrahedrally symmetric T-O vibrational stretching, are attributed to the Si-O tetrahedra in the zeolites [47]. The peaks at 1249 cm^−1^ and 514 cm^−1^ are characteristic of five-membered ring (5-MR) chains and different ring structures of zeolites [48]. Combining the above results, all the MCM-ZrP catalysts retain the characteristic functional groups of MCM-22.

The N_2_ adsorption/desorption isotherms of MCM-22 and pillared MCM-ZrP are illustrated in Figure 1c. MCM-22 exhibits a typical type I isotherm according to the IUPAC classification, showing a sudden increase in N_2_ adsorption at very low p/p_0_ values, attributed to the micropore structure of the material [49]. In contrast, the isotherms of MCM-ZrP form a clear H4-type hysteresis loop, indicating a type IV isotherm, which suggests a hierarchical porous system consisting of both micropores and mesopores [41]. These pore networks are effective for increasing the accessibility of active sites and the rate of intracrystalline diffusion, resulting in improved catalytic performances [50].

The textural properties of MCM-22 presented in Table 1 differ significantly from those of MCM-ZrP. Notably, after pillaring, the external surface area increased for MCM-ZrP2 (173 m^2^/g), MCM-ZrP1 (178 m^2^/g), and MCM-ZrP0.5 (153 m^2^/g), compared of the parent MCM-22 (78 m^2^/g). MCM-ZrP significantly reduces the catalyst’s micropore volume (from 0.199 cm^3^/g to about 0.05 cm^3^/g), likely because the interlayer ZrP pillars blocked some of the micropores or deposited on the surface of the zeolite crystal [51], resulting in a drastic reduction of the original micropores. The smaller increase in nitrogen adsorbed volume observed at low partial pressures for the MCM-ZrP sample can also be attributed to the partial blocking of micropores by the pillars.

**Figure 2:**
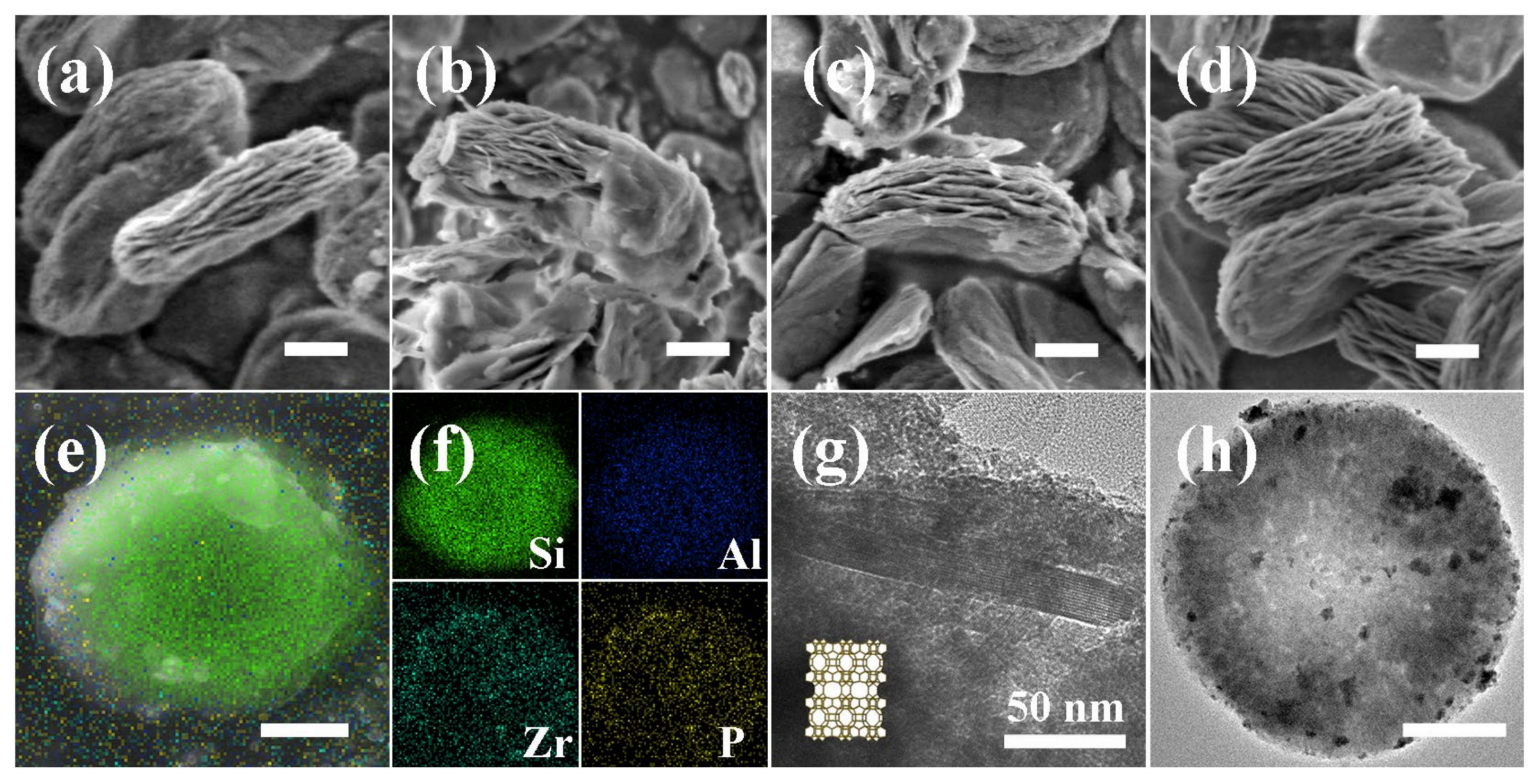
Scanning electron microscopy (SEM) images of (**a**) MCM-22, (**b**) MCM-ZrP0.5, (**c**) MCM-ZrP1, and (**d**) MCM-ZrP2. EDS elemental mappings (**e**,**f**) of Si, Al, Zr, and P of MCM-ZrP1 catalyst. TEM image of MCM-22 (**g**) and MCM-ZrP1 (**h**). The inset in (**g**) is the proposed structure of **MWW** topology. All unmarked scale bars in (**a**–**h**) are 1 µm. X in MCM-ZrPx catalysts represents the feeding molar ratio of Zr to P.

The morphology of MCM-22 and different MCM-ZrP samples is illustrated in Figure 2a–d. The MCM-22 material demonstrates a layered stacking morphology, characterized by a concave core enveloped by a convex outer boundary. This plate-like structure imparts distinctive geometric features to the material, with an estimated diameter ranging between 3.7 to 4 µm and a thickness spanning from 0.9 to 1.2 µm. MCM-ZrP2 appears almost identical to the parent MCM-22. In contrast, MCM-ZrP0.5 and MCM-ZrP1 show some extralaminar species, which we hypothesized to be a consequence of incomplete pillaring of the ZrP during the synthesis. Energy dispersive X-ray spectroscopy (EDS) results (Figure 2e,f) further confirm the good dispersion of Zr and P species.

Transmission electron microscopy (TEM) images of MCM-22 and MCM-ZrP1 are presented in Figure 2g and Figure 2h, respectively. Due to the relatively large size of the crystals, resolving the intricate details of the lattice structure proved challenging. Consequently, obtaining a clear image of the entire unit cell was not feasible. However, the lattice can be partially observed in the fragmented regions at the periphery of the particles. The inset in Figure 2g depicts a simulated image of the **MWW** framework for reference. Figure 2h shows a wider view of the ZrP1 sample. The noticeable contrast between the darker edges and the brighter center suggests the presence of a thin central region. Additionally, the image reveals the apparent aggregation of smaller particles, potentially indicative of imperfections during ZrP incorporation.

Table 2 presents elemental composition detected via inductively coupled plasma (ICP), revealing a Si/Al ratio ranging from approximately 10.5 to 12 for the pillared products. This range contrasts with the Si/Al ratio of 12.4 measured in the parent MCM-22, which closely resembled the initial feed ratio. We speculate that the slight decrease in the Si/Al ratio, despite remaining relatively close to the initial value, is due to the use of alkali during the swelling process. Additionally, the Zr/P ratios in MCM-ZrP0.5 and MCM-ZrP1 are relatively consistent with the feed ratio. In contrast MCM-ZrP2 has a slightly lower Zr/P ratio than expected, suggesting potential variations in the extent of ZrP formation, which might be influenced by the specific phosphorus dosage employed during synthesis. The observed Zr/Si ratios displayed a more pronounced deviation from the initial feed ratios. This significant disparity suggests a complex interplay between the amount of ZrP formed and the overall framework composition, likely mediated by the phosphorus content.

**Figure 3:**
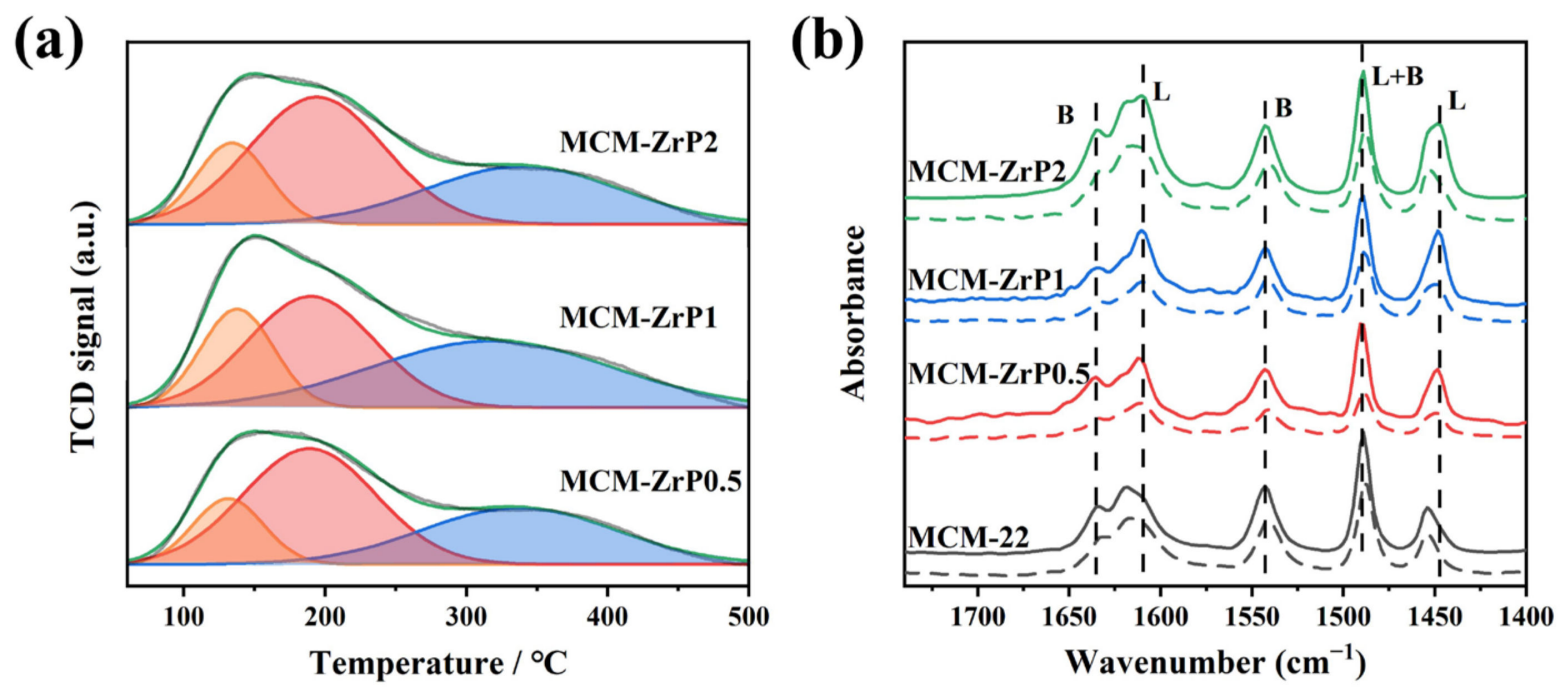
(**a**) NH_3_-TPD profiles and (**b**) Py-FT-IR spectra after pyridine adsorption by different catalysts at 150 °C (solid line) and 250 °C (dashed line). X in the MCM-ZrPx catalysts represents the feeding molar ratio of Zr to P. Weak acid sites: 130 °C (Orange) and 200 °C (Pink); Medium-strength acid sites: 340 °C (Blue).

Since acidity is a key physicochemical property affecting the catalytic conversion of biomass, we characterized the acidity of both the parent and pillared MCM-22. NH₃ temperature-programmed desorption (NH_3_-TPD) analysis (Figure 3a) was employed to investigate the acidic properties of three material compositions (Table 2). The concentration of acid sites was determined by the amount of desorbed ammonia, while the NH_3_ desorption peak temperature closely correlates with the strength of the acid sites [52]. The TPD curves of MCM-ZrP revealed a predominance of weak and medium-strength acid sites, as determined by peak deconvolution analysis (Figure 3a). These weaker and moderately strong acidic functionalities constitute the majority of the acidic character of MCM-ZrP. The presence of medium-strength acid sites is evidenced by the NH₃ desorption peak observed at 340 °C (Blue). Additionally, two distinct peaks at lower desorption temperatures (130 °C (Orange) and 200 °C (Pink)) suggest the existence of a population of weak acid sites within the material.

The total acidity of MCM-22 was 543 μmol/g, while MCM-ZrP1 had the highest acidity of 976 μmol/g. The acidity of MCM-ZrP0.5 (815 μmol/g) and MCM-ZrP2 (943 μmol/g) were also much higher than that of the parent MCM-22, suggesting that the pillaring of ZrP significantly enhanced the total acidity. Interestingly, pillared catalysts exhibited a marked increase in the abundance of weak acid sites. While the concentration of medium-strength acid sites in MCM-ZrP1 experienced a notable enhancement, those in MCM-ZrP0.5 and MCM-ZrP2 remained largely comparable to their parent counterparts. This variation in acid site concentration might be attributable to the presence of different ZrP species, potentially influenced by the Zr/P ratio employed during synthesis. Tailoring the Zr/P ratio could offer a means to manipulate the concentration and strength of acid sites within these materials.

Pyridine-adsorbed FT-IR (Py-FT-IR) was utilized to quantitatively analyze the Brønsted and Lewis acid sites of the catalysts (Figure 3b). Peaks at 1490, 1540, and 1635 cm⁻^1^ correspond to pyridine adsorbed on Brønsted acid sites, including those associated with tetrahedral framework Al and P-(OH)_n_ species [53,54]. In contrast, Lewis acid sites involving Zr species are indicated by pyridine adsorption peaks at 1450, 1490, and 1610 cm⁻^1^ [55]. Typically, the relative abundance of Brønsted and Lewis acid sites within a catalyst can be determined by the ratio of peak integral areas at about 1540 and 1450 cm⁻^1^, as reported in Li and Long’s work [56] and detailed in Table 2.

A comprehensive analysis of NH_3_-TPD and Py-FT-IR collected at 150 °C reveals that Brønsted acid sites (399 μmol/g) account for over 70% of the total acidity within the MCM-22 material. The addition of ZrP resulted in a significant increase in the proportion of Lewis acid in the catalyst, with the order being MCM-ZrP1 (520 μmol/g) > MCM-ZrP2 (445 μmol/g) > MCM-ZrP0.5 (286 μmol/g). Additionally, a notable increase in the Brønsted acid content of MCM-ZrP materials was observed, with values ranging from approximately 50 to 130 μmol/g. This suggests that the incorporation of ZrP not only affects the framework properties but also enhances the acid sites within the material. Based on reports, we assume that the Zr/P ratio influences the different phosphorus species in the Zr-O-P bonds, leading to a significant difference in the amount of Lewis and Brønsted acid [57]. Furthermore, pyridine-adsorbed FT-IR collected at 250 °C shows that all materials retain both Lewis and Brønsted acidity at high temperatures, representing their medium-strength acidic nature.

### 2.2. Screening of Catalysts

**Scheme 2:**
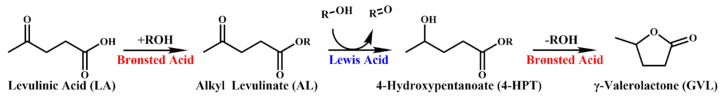
Proposed catalytic reaction pathway of LA to GVL.

**Figure 4:**
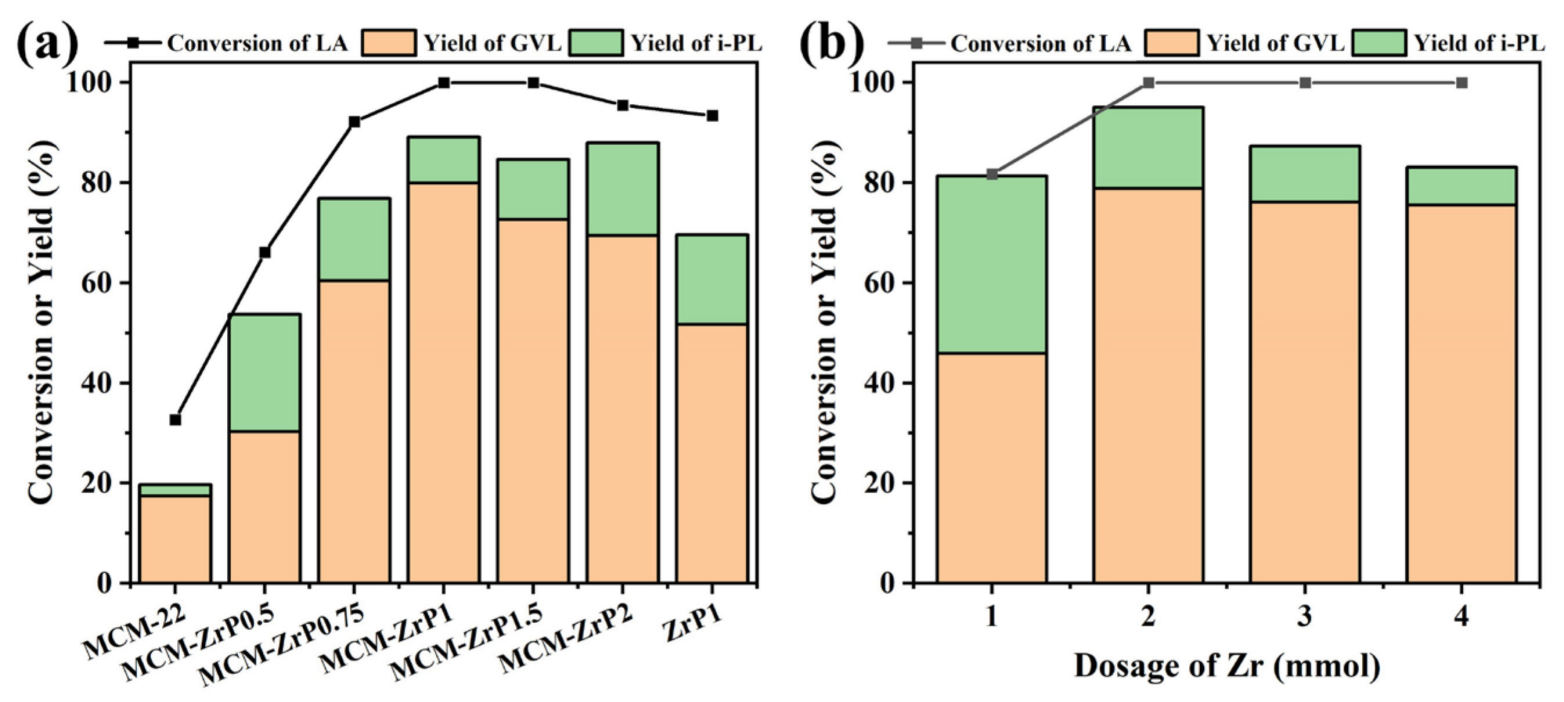
Catalytic performance of (**a**) different catalysts; (**b**) different dosages of Zr in MCM-ZrP1. Conditions: LA (33 mmol/L), IPA (5 mL), catalyst (30 mg), 160 °C, 6 h. X in the MCM-ZrPx catalysts represents the feeding molar ratio of Zr to P.

Following characterization, we conducted an initial screening of various catalysts, with the results shown in Figure 4. The catalytic evaluation was carried out at 160 °C using isopropyl alcohol (IPA) as a hydrogen source. As shown in Scheme 2, Lewis acid has been reported to be responsible for the transfer hydrogenation of LA and its esters, while Brønsted acid is involved in the esterification of LA and the transesterification reaction [54,58,59]. The parent MCM-22 exhibited low conversion of LA and a low yield for GVL, likely due to its weak Lewis acidity. Similarly, although the conversion of LA was high with ZrP1 catalysis, the yield of GVL was only about 50%, attributed to its low specific surface area and limited contact between the acidic sites and the substrate.

We investigated the impact of the Zr/P ratio on GVL yield. All the MCM-ZrP samples showed high LA conversion in 6 h (>90%), except for MCM-ZrP0.5. However, the yield of GVL initially increased as the Zr/P ratio increased from 0.5 to 1. Beyond this point, further increase in the Zr/P ratio led to a decrease in GVL yield, with MCM-ZrP1 demonstrating the optimal performance. Interestingly, the yield of isopropyl levulinate (i-PL), the esterification product of LA, exhibited a trend diametrically opposed to that observed for GVL. Our findings suggest a non-monotonic relationship between the Zr/P ratio and Lewis acid. Initially, the decreases in Zr/P ratio from 2 to 1 led to a gradual enhancement in both Lewis acidity and GVL yield. However, when the Zr/P ratio fell below 1, the concentration of Lewis acid sites dropped significantly, matching our measurements and coinciding with a dramatic decline in GVL yield. Concurrently, the concentration of Brønsted acid sites also depends on the phosphorus content. Our findings indicate that MCM-ZrP1 exhibits the optimal balance of Lewis and Brønsted acidity among the materials studied.

Furthermore, the combination of ZrP1 and MCM-22 significantly increased the yield of GVL. The incorporation of ZrP enhances the catalytic process through two key mechanisms—(a) the increase in the concentration of Lewis and Brønsted acid sites plays a crucial role in the catalytic conversion of LA, and (b) the introduction of ZrP results in a higher specific surface area for the catalyst, facilitating improved contact between the substrate and the catalyst, ultimately enhancing the overall reactivity of the system.

Subsequently, the amount of Zr doping was also considered. We maintained a constant Zr/P ratio and explored the effect of varying Zr doping amount. As the amount of Zr doping increased from 1 mmol to 4 mmol, the GVL yield initially increased and then decreased. We speculate that the decrease is due to the high content of ZrP, which blocked some active sites. Based on these findings, MCM-ZrP1 will be employed as the catalyst for further investigations.

### 2.3. Catalytic Performance of Catalysts

**Figure 5:**
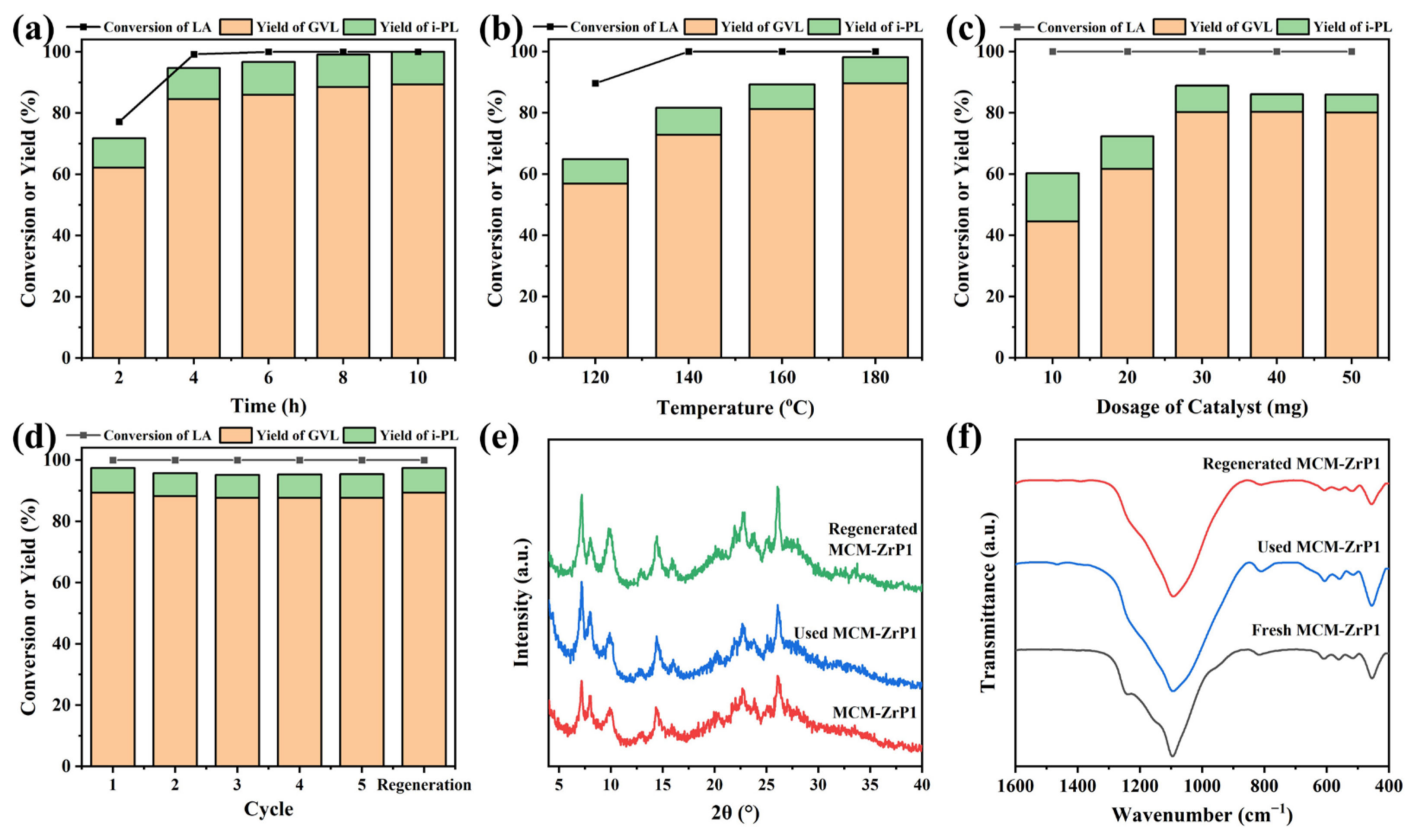
Catalytic performance of (**a**) different times at 160 °C, (**b**) different temperatures for 4 h, (**c**) different dosages of catalyst at 160 °C for 6 h, (**d**) recycling test of MCM-ZrP1, (**e**) XRD patterns, and (**f**) FT-IR spectra of fresh, used, and regenerated MCM-ZrP1. Conditions: LA (33 mmol/L), IPA (5 mL), catalyst (30 mg). X in the MCM-ZrPx catalysts represents the feeding molar ratio of Zr to P.

After screening the catalysts, we optimized the reaction conditions (Figure 5). First, we optimized the reaction time by conducting experiments at 160 °C for varying durations. As anticipated, the conversion of the catalytic reaction approached 99.9% within 4 h, and the yield of GVL gradually increased over time, reaching 89.4% at 8 h. Beyond this point, the yields of both final products and intermediates plateaued, indicating that equilibrium was achieved.

Next, we investigated the influence of temperature at a fixed reaction time of 4 h. At 140 °C, complete conversion (99.9%) of LA was achieved, but the combined yield of the desired product (GVL) and the intermediate (i-PL) remained low, reaching only 75%. This discrepancy suggests potential adsorption of LA onto the catalyst, hindering further conversion. In contrast, at 180 °C, LA conversion reached 99.9% rapidly within 4 h, with a significantly higher GVL yield of 89.6%. These results clearly demonstrate a temperature-dependent reaction. Increasing the temperature promotes LA conversion and GVL yield, indicating a strong influence of temperature on the reaction pathway. Notably, the yield of the intermediate product, i-PL, remained relatively constant at around 8%. This observation suggests a potential link to the initial concentration of LA and warrants further exploration.

The catalyst dosage is an important factor to consider (Figure 5c). Under the conditions of 160 °C for 6 h, increasing the catalyst dosage from 10 mg to 30 mg resulted in a significant increase in GVL yield (from 44.5% to 80.2%), accompanied by a slight decrease in i-PL yield (from 19.1% to 8.6%). However, when the catalyst dosage exceeded 30 mg, the GVL yield showed only minimal changes, indicating that 30 mg represents the optimal catalyst loading for this reaction.

Catalyst reusability is a critical parameter for practical applications. We subjected the catalysts to multiple catalytic cycles to assess their reusability. After each cycle, the catalyst was rinsed with isopropanol, dried, and reused. Notably, after five cycles, the catalytic conversion remained exceptionally high at 99.9%, with only a slight decrease in GVL yield. This decrease is likely due to the formation of carbonaceous deposits, known as humins, during the reaction process. These humins can accumulate within the catalyst pores, hindering substrate diffusion and ultimately reducing yield. After the fifth cycle, the catalyst was separated, calcined, and regenerated.

A key advantage of zeolite catalysts is their structural stability, which allows for high-temperature calcination to remove humin deposits. We observed that post-cycle calcination effectively restored GVL yield to near-initial levels, indicating the successful regeneration and reusability of the acidic sites within the catalyst. To further investigate the impact of recycling, we performed characterization of the fresh, used, and regenerated catalyst samples. The XRD patterns (Figure 5e) indicate that the used catalyst retains the same structural features as the original MCM-ZrP1, with no significant peak intensity loss. The regenerated sample also maintains structural integrity, suggesting excellent catalyst stability and potential for reuse. FT-IR analysis (Figure 5f) was used to assess potential structural changes. The FT-IR spectra of the regenerated and used samples, compared to the fresh catalyst, revealed minimal structural degradation. This suggests that the decline in catalytic performance is likely due to carbonaceous species accumulating on the catalyst surface. This observation underscores the excellent recyclability and reusability of the synthesized catalyst.

### 2.4. Discussion of Catalytic Mechanism

**Figure 6:**
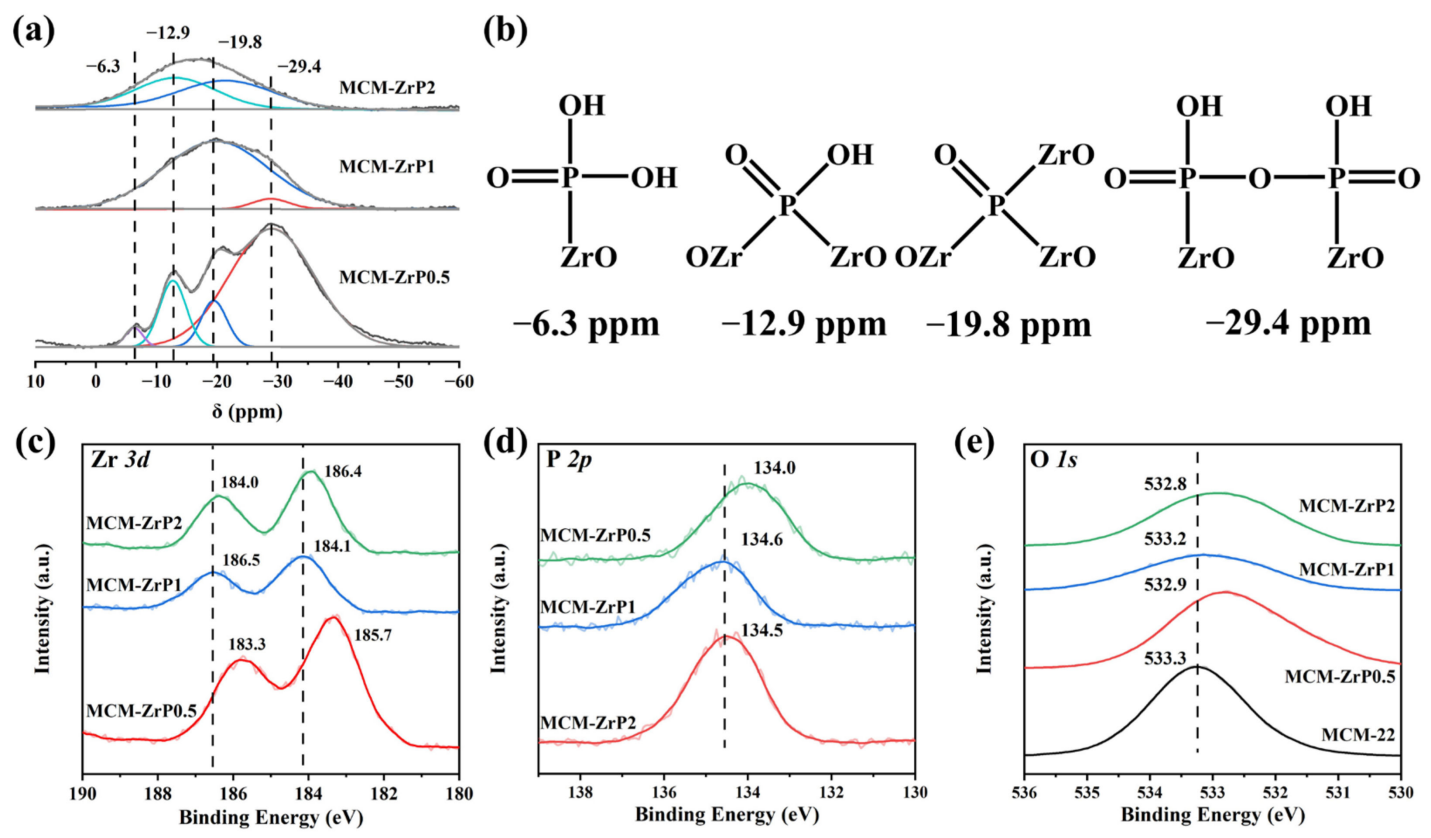
(**a**) ^31^P MAS NMR spectra of the catalysts and the deconvolution of the signals (Purple line: −6.3 ppm; Cyan line: −12.9 ppm; Blue line: −19.8 ppm; Red line: −29.4 ppm), (**b**) proposed structure of phosphate species in the catalysts, and high-resolution XPS spectra of the (**c**) Zr *3d*, (**d**) P *2p*, and (**e**) O *1s* regions for the catalysts. X in the MCM-ZrPx catalysts represents the feeding molar ratio of Zr to P.

Solid-state ^31^P magic angle spinning (MAS) nuclear magnetic resonance (NMR) was employed to determine the phosphorus species present in different MCM-ZrP catalysts (Figure 6a). This characterization method is useful for identifying the coordination states of phosphorus atoms in the bulk phase. A chemical shift to more negative values indicates both an increase in the number of P-O-Zr bonds [60,61] and an increase in the chain length of the phosphorus atoms [62].

The MCM-ZrP catalysts predominantly exhibit four phosphorus species, with the proposed structures presented in Figure 6b. The resonance peaks at −6.3, −12.9, and −19.8 ppm are attributed to tetrahedral phosphates bonded to one zirconia group and two hydroxyl groups (Zr-O)_1_-PO(OH)_2_, two zirconia groups and one hydroxyl group (Zr-O)_2_-PO(OH)_1_, and three zirconia groups (Zr-O)_3_-PO, respectively. The high-intensity peak at -29.4 ppm suggests relatively large amounts of polyphosphates (P-O-P), due to the condensation of different phosphate species [63,64]. All the samples showed relatively broad peaks, indicative of their amorphous nature [65].

By comparing the peaks, we found that the P species in MCM-ZrP1 and MCM-ZrP2 are similar but present in different quantities. The P atoms in MCM-ZrP1 primarily exhibit a large amount of (Zr-O)_3_-PO and a trace amount of polyphosphate, which may be the key to the standout performance of MCM-ZrP1. Numerous studies have shown that phosphorus atoms coordinated to one (δ = ~−5 ppm), two (δ = ~−12 ppm), and three (δ = ~−20 ppm) zirconia groups are responsible for generating Brønsted acid sites [62,64,65]. Polyphosphates have also been reported to exhibit high Brønsted acidity, attributed to the condensation and deprotonation of phosphate species. The P-O-P linkages in polyphosphates can extract electrons from the terminal phosphate groups, thus enhancing the acidity of the surface P-OH groups [66]. Regardless of whether it was MCM-ZrP0.5, dominated by polyphosphate, or MCM-ZrP1 and MCM-ZrP2, dominated by di- and tri-coordinated zirconia groups, the Brønsted acid acidity increased compared to the parent MCM-22.

Meanwhile, solid-state ^31^P MAS NMR data and the proposed structural model support our initial hypothesis regarding Lewis acidity. Notably, the increased concentration of (Zr-O)_3_-PO upon decreasing the Zr/P ratio from 2 to 1 appears critical for generating more accessible Lewis acid sites. MCM-ZrP1 appears to possess a greater number of “open” zirconium sites, which are hypothesized to be the key factor behind its exceptional catalytic performance [67,68]. We speculate that in (Zr-O)_3_-PO species, two of the zirconia groups are responsible for connecting with the silica hydroxyl groups in the zeolite MCM-22, while the third zirconia group plays a decisive role in the catalytic reaction. This observation aligns with the enhanced catalytic activity measured. We propose that Lewis acidity in ZrP primarily originates from tri-coordinated Zr species. Consistent with this proposal, MCM-ZrP1, possessing the highest concentration of these Zr sites, exhibits the strongest Lewis acidity. MCM-ZrP2 follows this trend, while MCM-ZrP0.5, with the lowest concentration of tri-coordinated Zr, demonstrates the lowest concentration of Lewis acid sites.

In addition to ^31^P MAS NMR, we used X-ray photoelectron spectroscopy (XPS) to determine the species composition of the catalysts and study the intensity of the active sites in different samples. The Zr, P, and O XPS spectra of various samples are shown in Figure 6c–e. The Zr *3d* spectra mainly consist of two peaks, Zr *3d_5/2_* at about 184 ev and Zr *3d_3/2_* at about 186 ev, which are characteristic peaks of tetravalent Zr [69]. XPS analysis of the Zr *3d* peaks revealed a slight shift of approximately 0.8 eV towards lower binding energy for MCM-ZrP0.5 compared to MCM-ZrP1 and MCM-ZrP2. This observation signifies higher binding energies for Zr in MCM-ZrP1 and MCM-ZrP2, indicative of a more positively charged state for the Zr atoms. Consequently, the Zr centers in these materials exhibit enhanced oxygen affinity and Lewis acidity [70].

Similarly, the typical P *2p* binding energy is 134.5 eV, characteristic of pentavalent tetra-coordinated phosphorus [71]. For different catalysts, the higher P *2p* binding energy results from the increased polarity of the surface P-O bonds, which can be enhanced by hydroxyl groups bound to the phosphorus atoms, suggesting an increase in the number of acidic sites on the surface. Additionally, the O *1s* peak has a higher binding energy, indicating that the oxygen atoms have more negative charge and an increased number of surface hydroxyl groups [72].

### 2.5. Extended Applications in Cascade Biomass Conversions

**Figure 7:**
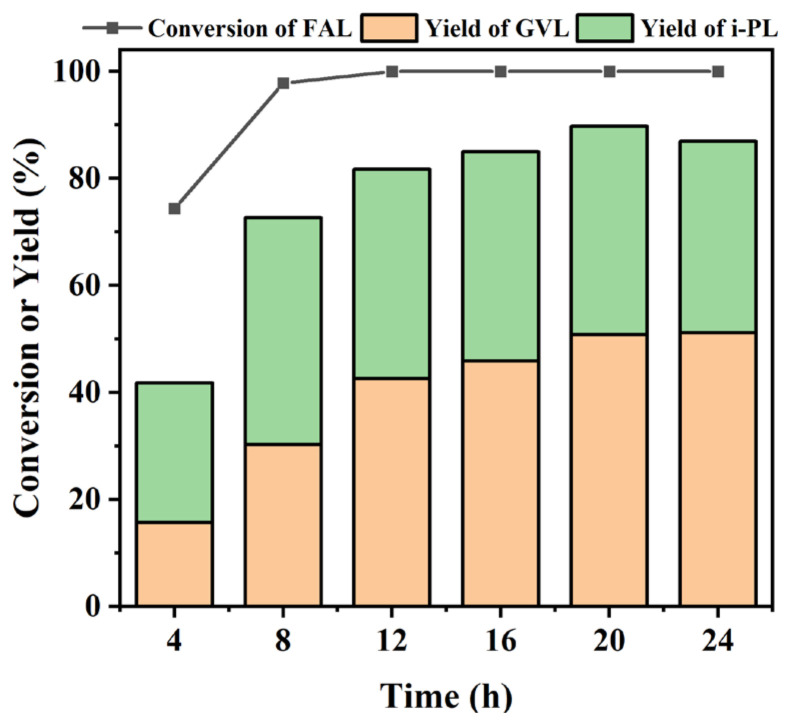
Catalytic performance of FAL to GVL and i-PL in different time of MCM-ZrP1. Conditions: FAL (33 mmol/L), IPA (5 mL), catalyst (30 mg), 160 °C. X in the MCM-ZrPx catalysts represents the feeding molar ratio of Zr to P.

Based on the catalytic performance of MCM-ZrP1, we aimed to extend the catalytic system to facilitate the one-pot generation of GVL starting from furfural (FAL) in Figure 7. This cascade conversion process is synergistically catalyzed by Lewis and Brønsted acids and involves the CTH of FAL and LA, as well as the ring-opening reaction of furfuryl alcohol to LA. This process generates a large number of byproducts, such as humins. The reaction achieved complete conversion (99.9%) of furfural within 20 h at 160 °C. While the GVL yield steadily increased, reaching 51.1% at 24 h, a significant portion (over 86%) was already formed after 20 h. Minimal byproduct generation was observed, highlighting the effectiveness of the mesoporous structure in the MCM-ZrP1 catalyst.

## 3. Materials and Methods

### 3.1. Materials

Fumed silica (Xuzhou Tiancheng Chlor_alkali Co., Ltd., Xuzhou, China), sodium hydroxide (Beijing Chemical Works, Beijing, China), sodium nitrate (Beijing Chemical Works, Beijing, China), hexamethyleneimine (HMI, Aladdin Industrial Corporation, Shanghai, China), zirconium oxychloride (Aladdin Industrial Corporation), ammonium dihydrogen phosphate (Tianjin Guangfu Fine Chemical Research Institute, Tianjin, China), ammonium chloride (Sinopharm Chemical Reagent Company, Beijing, China), cetyltrimethylammonium bromide (CTAB, Sinopharm Chemical Reagent Company, Beijing, China), and tetrapropylammonium hydroxide (TPAOH, 40% solution in water, Zhejiang Kente Chemical, Hangzhou, China) were used as received. Levulinic acid (LA, 98%), levulinate esters (>98%), and γ-valerolactone (GVL, 98%) were purchased from TCI Chemical Reagent Company (Shanghai, China). Isopropyl alcohol (IPA, AR, ≥99.7%) were obtained from Sinopharm Chemical Reagent Company (Shanghai, China). Thick-walled pressure-resistant bottles were purchased from Beijing Xavier Glass Instruments Co., Beijing, China.

### 3.2. Catalyst Preparation

#### 3.2.1. Synthesis of MCM-22 (P)

Following the procedures reported in the literature [73,74], MCM-22 (P), where P represents the MCM-22 precursor, was synthesized from a mixed solution with a molar composition of 0.18 NaOH:SiO_2_:0.033 Al_2_O_3_:0.5 HMI:20 H_2_O. First, 0.116 g of sodium hydroxide and 0.234 g of sodium meta-aluminate (41% Na_2_O, 48% Al_2_O_3_) were dissolved in deionized water. Then, 1.65 g of HMI was added drop by drop as a template. Following this, 2.0 g of fumed silica was added into under vigorous stirring, and stirring continued until the silica was completely dissolved. The resulting homogeneous gel was then transferred to a PTFE-lined stainless-steel reactor and reacted for 5 d at 150 °C under static conditions. The product was separated by filtration, washed several times with deionized water, and dried overnight.

#### 3.2.2. Swelling and Pillaring

MCM-22(P) was swollen with CTAB at ambient temperature under high-pH conditions. The composition of the swelling mixture followed the methods reported by Kresge [75] and Corma [51]. Typically, 9.0 g of aqueous slurry of MCM-22(P) (20 wt % solids) was mixed with 35.0 g of 29 wt % CTAB aqueous solution and 11.0 g of an aqueous solution of 40 wt % TPAOH. The pH of the resulting mixture was approximately 13.5. The mixture was stirred for 16 h at ambient temperature, and the swollen materials were then washed with distilled water through successive centrifugation cycles (10,000 rpm) up to ten times and dried overnight at ambient temperature.

The pillaring process was modified based on the literature reported by Barth et al. [42]. The alumina pillaring agent was prepared by adding an NH_4_H_2_PO_4_ solution (100 mL, with varying concentrations of 0.1 M, 0.2 M, or 0.4 M) dropwise to a ZrOCl_2_ solution (100 mL, 0.2 M) under stirring. The mixture was stirred at 80 °C for 4 h and then aged for 48 h at ambient temperature. The pillaring process was carried out by adding the swollen MCM-22 precursor (1 g in a 20 wt % water suspension) to the pillaring solution (approximately 2 mmol Zr/g swollen MCM-22 precursor). The mixture was stirred at ambient temperature for 12 h. The product was filtered, dried overnight at ambient temperature, and then calcined in air at 550 °C (2 °C/min) for 6 h. The catalyst was designated as MCM-ZrP_x_, where x represents the Zr/P feeding ratio.

All samples were prepared by performing liquid-phase ion exchange twice with a 1M NH_4_Cl solution for 4 h at 80 °C, followed by calcination at 550 °C (2 °C/min) for 6 h.

### 3.3. Characterization

XRD was performed using a Bruker D8 Advance (Bruker AXS, Karlsruhe, Germany) diffractometer with Cu-Kα radiation. The X-ray source wavelength was set at 0.154 nm, with a step width of 0.02° and a scanning speed of 10° per minute, covering a 2*θ* range of 4° to 40° The Brunauer–Emmett–Teller (BET) surface area was measured using an BSD-VD12 Vacuum Degassing Instrument (BSD Instrument, Beijing, China). NH_3_-TPD was conducted on a Micromeritics AutoChem II 2920 automated chemisorption analyzer (Micromeritics, Norcross, GA, USA) to assess the surface acidity of the catalysts. XPS measurements were carried out with an ESCALAB250 photoelectron spectrometer (Thermo Scientific, Waltham, MA, USA) equipped with a charge neutralizer and a Mg Kα X-ray source. The morphology of the samples was investigated using TEM (Tecnai F20, FEI, Hillsboro, OR, USA) and SEM (Jeol JSM-7800 F, Akishima-shi, Japan). The elemental compositions of the catalysts were determined by ICP using a Thermo Scientific iCAP 7600 ICP-OES (Waltham, MA, USA). FT-IR spectra were recorded between 400 to 4000 cm^−1^ using a Bruker VERTEX 80 V spectrometer (Billerica, MA, USA). ^31^P MAS NMR spectra were obtained on a Bruker Avance NEO 600M (Karlsruhe, Germany) system with 14.09 T magnetic field. Ammonium dihydrogen phosphate was used as the standard samples of ^31^P, with a calibration of 4.1 ppm. Py-FT-IR tests were conducted at ambient temperature in air using a JASCO FT/IR-410 (Tokyo, Japan) with a KBr dispersion of a 420 Herschel series. Samples were processed at 250 °C for 1 h, then ramped to 150 °C and 250 °C at a rate of 10 °C/min for testing. The concentration of the Brønsted and Lewis acid sites was calculated via the following equations:

$$C_{B}\left(\mu mol\cdot g^{-1}\right)=\frac{1.88\times IA_{B}\times R^{2}}{W}$$

$$C_{L}\left(\mu mol\cdot g^{-1}\right)=\frac{1.42\times IA_{L}\times R^{2}}{W}$$

where C_B_ and C_L_ stand for the concentration of Brønsted and Lewis acid sites, respectively; IA_B_ and IA_L_ stand for the integrated absorbance peak at 1540 cm^−1^ for Brønsted acid sites and the peak at 1450 cm^−1^ for Lewis acid sites, respectively; *R* stands for the radius of the self-supported disk; and *W* stands for the weight of the sample.

### 3.4. Experiment of Hydrogen Transfer Reactions of Biomass-Derived Carbon Oxides and Sample Analysis

The reaction was conducted in 15 mL thick-walled, pressure-resistant bottles. In a typical experiment, LA (33 mmol/L), catalysts (30 mg), and IPA (5 mL) were added to the bottle and sealed. The bottle was heated to the desired temperature (120–180 °C) with vigorous stirring (600 rpm) for a specified duration (1–10 h). After the reaction, the reactor was quickly cooled to ambient temperature. The reaction solution, after the catalyst was removed by filtration, was analyzed using a TRACE ISQ GC-MS (Thermo Scientific Co., Waltham, MA, USA) with a TR-WAX-MS column (Thermo Scientific Co., Waltham, USA, 30.0 m × 320 µm × 0.25 µm). The temperature program started at 60 °C for 1 min, then increased from 60 °C to 230 °C at a rate of 15 °C/min and held for 2 min. The conversion of LA as well as the yield and selectivity of GVL were defined according to previous reports [76]. The formulae for calculating conversion of LA and yield of i-PL/GVL are as follows:

$$Conversion\left(FAL\right)=\left(1-\frac{Mole\;of\;LA}{Initial\;mole\;of\;LA}\right)\times 100\%$$

$$Yield\left(i-PL/GVL\right)=\frac{Mole\;of\;i-PL/GVL}{Initial\;mole\;of\;i-PL/GVL}\times 100\%$$

For product analysis, the concentrations of each component in the mixtures were quantified using the external standard method to minimize errors.

## 4. Conclusions

In summary, we synthesized a novel zeolite MCM-36 derivative using zirconium phosphate as a pillar support to create a catalytically active composition. Unlike traditional oxides, the incorporation of zirconium phosphate endowed the catalyst with both Lewis and Brønsted acidity, enhancing the conversion and selectivity in the catalytic conversion of levulinic acid (LA) to γ-valerolactone (GVL). By adjusting the ratio, we achieved a 99.9% conversion of LA within 4 h, and the yield of GVL peaked at 89.6% after 8 h. We further investigated the phosphorus species in depth using ^31^P MAS NMR to determine the structure of catalysts with different Zr/P ratios. We observed higher catalytic activity for phosphate species with phosphorus coordinated to two and three zirconium oxide groups (with chemical shifts of −12.9 ppm and −19.8 ppm, respectively). Additionally, we extended the application of the catalyst to the one-pot catalytic conversion of furfural (FAL) to GVL, achieving a 99.9% conversion of FAL and an overall yield of 79.4% for GVL and isopropyl levulinate (i-PL) in 8 h. These results indicate that the catalyst has significant potential for application in biomass conversion reactions, offering a sustainable chemical conversion process for biomass energy.

## Figures and Tables

**Table 1:** Textural and structural properties of MCM-22 and the MCM-ZrPx catalysts, where x represents the feeding molar ratio of Zr to P.

	S_BET_ ^a^ (m^2^/g)	S_micro_ ^b^ (m^2^/g)	S_ext_ ^b^ (m^2^/g)	V_total_ ^c^ (cm^3^/g)	V_micro_ ^b^ (cm^3^/g)
MCM-22	581	503	78	0.796	0.199
MCM-ZrP0.5	298	145	153	0.635	0.064
MCM-ZrP1	286	108	178	0.612	0.046
MCM-ZrP2	306	133	173	0.427	0.056

^a^ Surface area was determined by BET method; ^b^ Micropore surface area, external surface area, and micropore volume were determined by t-plot; ^c^ Total pore volume at p/p_0_ = 0.99 cm^3^·g^−1^.

**Table 2:** Elemental analysis and concentration of acid sites for different catalysts. X in the MCM-ZrPx catalysts represents the feeding molar ratio of Zr to P.

	Si/Al ^a^	Si/Zr ^a^	Zr/P ^a^	Acidic Sites (μmol/g)			B/L ^c^
				Weak ^b^	Moderate ^b^	Total ^b^	
MCM-22	12.40	-	-	241	301	543	2.76
MCM-ZrP0.5	10.42	4.75	0.63	527	287	815	1.85
MCM-ZrP1	10.88	3.51	1.11	560	417	977	0.88
MCM-ZrP2	12.15	15.89	1.57	634	309	943	1.12

^a^ molar ratios of different elements were determined by ICP; ^b^ the amount of acid sites was determined by NH_3_-TPD; ^c^ the number of Brønsted/Lewis acidic sites was calculated by Py-FT-IR.

## Data Availability

Data are contained within the article.
